# Delayed Vaginal Cuff Ectopic Pregnancy Following Hysterectomy: A Case Report

**DOI:** 10.1155/crog/5973350

**Published:** 2026-04-26

**Authors:** Emmanuel Nshimiyumuremyi, Dawit Worku Kassa, Bagambe Patrick, Diomede Ntasumbumuyange, Fikremelekot Temesgen, Thomas Ugiruwatuma, Viateur Mutabazi, Theogene Bazimaziki, Emmanuel Itangishaka, Vedaste Musangamfura, Etienne Nsengimana, Oscar Umuhoza

**Affiliations:** ^1^ Department of Obstetrics and Gynecology, Kigali University Teaching Hospital (KUTH), Kigali City, Rwanda; ^2^ Department of Obstetrics and Gynecology, Muhima District Hospital, Kigali City, Rwanda; ^3^ Obstetrics and Gynecology Residency Program, University of Rwanda, Kigali City, Rwanda, ur.ac.rw

**Keywords:** case report, ectopic pregnancy, extrauterine pregnancy, laparotomy, (MeSH), posthysterectomy pregnancy, vaginal cuff ectopic pregnancy

## Abstract

**Background:**

Vaginal cuff ectopic pregnancy after hysterectomy is extremely rare and poses diagnostic challenges due to unusual anatomical implantation and low clinical suspicion. Delay in diagnosis may result in catastrophic hemorrhage.

**Case Presentation:**

A 30‐year‐old G7P5014 (gravida 7 para 5 1 miscarriage and 4 living children, one stillbirth because of uterine rupture) woman presented 10 months after total abdominal hysterectomy and right oophorectomy with nausea and dizziness. Transvaginal ultrasound revealed a live 8‐week embryo. She initially declined surgery. Two days later, she returned with abdominal pain, hypotension (BP 90/70 mmHg), and tachycardia (HR 130 bpm). Repeat ultrasound showed intra‐abdominal free fluid. Emergency laparotomy revealed 2.5‐L hemoperitoneum and a gestational sac implanted on the vaginal cuff. The sac was excised, hemostasis secured, and the patient recovered well after transfusion.

**Conclusion:**

Clinicians should maintain high suspicion for ectopic pregnancy in reproductive‐aged women with preserved ovaries, even after hysterectomy. Early diagnosis and urgent surgical intervention are essential to prevent mortality.

## 1. Introduction

Ectopic pregnancy is a potentially life‐threatening condition in which implantation occurs outside the uterine cavity and remains an important cause of early pregnancy‐related maternal morbidity and mortality [1]. Although the majority of ectopic pregnancies occur in the fallopian tubes, rare implantation sites such as the abdominal cavity, cervix, cesarean scar, and vaginal cuff have been described [1–3]. Posthysterectomy ectopic pregnancy is exceptionally uncommon, but it should not be overlooked in reproductive‐aged women with retained ovarian function who present with abdominal pain, dizziness, or a positive pregnancy test after hysterectomy [4].

Several mechanisms have been proposed to explain pregnancy after hysterectomy, including an unrecognized early pregnancy at the time of hysterectomy, transperitoneal migration of sperm or a fertilized ovum through a retained fallopian tube, and the presence of a vaginal cuff–peritoneal fistulous tract [4–6]. In delayed presentations occurring months after hysterectomy, transperitoneal fertilization and implantation at a susceptible scarred surface are considered the most plausible explanations [4–6]. Because implantation at the vaginal cuff is highly vascular and anatomically unusual, diagnosis may be delayed, and catastrophic hemorrhage can occur if the condition is not recognized promptly [4].

We report a rare case of a viable 8‐week ectopic pregnancy implanted at the vaginal cuff 10 months after total abdominal hysterectomy with right oophorectomy. This case highlights the importance of maintaining clinical suspicion for ectopic pregnancy even after hysterectomy, the diagnostic value of transvaginal ultrasound (TVUS) in altered pelvic anatomy, and the need for timely surgical intervention when clinical deterioration occurs.

## 2. Patient Information

A 30‐year‐old woman, gravida 7 para 5 abortion 1 and 4 living children (G7P5014), presented to Kigali University Teaching Hospital on June 11, 2025, with complaints of nausea and dizziness Table 1.

**Table 1:** Timeline.

Date	Event
August 2024	Total abdominal hysterectomy with right oophorectomy for postpartum hemorrhage secondary to uterine rupture
June 11, 2025	Presentation with nausea/dizziness; positive home pregnancy test
June 12, 2025	Hospital evaluation pregnancy test positive, transabdominal ultrasound noncontributory, transvaginal ultrasound identified live 8‐week embryo at vaginal cuff
June 12, 2025	Counseling for surgical management, patient initially declined intervention.
June 14, 2025	Re‐presentation with acute abdominal pain, hypotension (BP 90/70), tachycardia (HR 130); ultrasound revealed free fluid
June 14, 2025	Emergency laparotomy performed; 2.5‐L hemoperitoneum evacuated; ectopic gestational sac removed
June 14, 2025	Emergency explorative laparotomy, evacuation of 2.5‐L hemoperitoneum, excision of vaginal cuff ectopic pregnancy.
Postoperative (Day 0–3)	Hemodynamic stabilization with transfusion (4PRBC, 4FFP, 4 platelets): monitored in surgical ward.
Postoperative (Day 4–7)	Clinical recovery with stable vital signs, no evidence of ongoing bleeding or infection.
Follow‐up (2–4 weeks	Patient asymptomatic, normal recovery, no complications reported

She had undergone a total abdominal hysterectomy with right oophorectomy in August 2024 for postpartum hemorrhage secondary to uterine rupture.

Ten months later, she performed a home pregnancy test, which was positive, prompting her to seek medical consultation.

## 3. Clinical Findings

At presentation, the patient was hemodynamically stable and reported nonspecific symptoms, including nausea and dizziness, without abdominal pain or tenderness on examination.

A transabdominal ultrasound performed on June 12, 2025, demonstrated no intra‐abdominal free fluid and no identifiable intrauterine or adnexal gestation, consistent with the patient′s prior hysterectomy.

**Figure 1:**
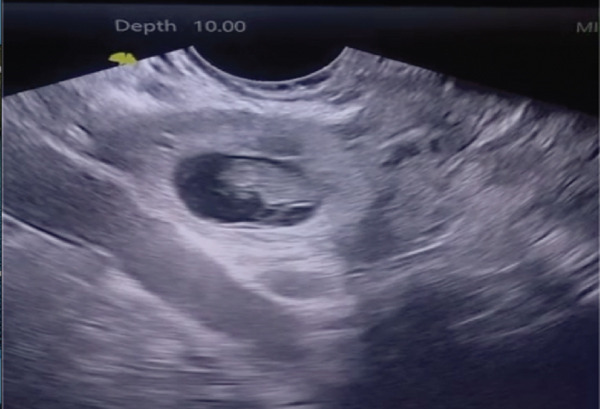
Transvaginal ultrasound showing a gestational sac with a live embryo.

Subsequent TVUS (Figure 1) provided detailed characterization of the ectopic implantation and revealed the following:

• A well‐defined gestational sac implanted at the vaginal cuff, located at the superior aspect of the vaginal vault
• The sac‐to‐cuff interface was clearly visualized, with the gestational sac embedded within the fibrous tissue of the cuff, without extension into surrounding pelvic organs
• A live embryo with a crown–rump length consistent with 8 weeks of gestation, with positive fetal cardiac activity
• No identifiable residual cervical canal, supporting true posthysterectomy anatomy
• Color Doppler imaging demonstrated increased peritrophoblastic vascularity surrounding the gestational sac, consistent with active trophoblastic invasion
• There was no sonographic evidence of deep invasion into adjacent structures such as the bladder or bowel
• Importantly, no fistulous tract was visualized between the vaginal cuff and the peritoneal cavity or adjacent organs

These findings were highly suggestive of a vaginal cuff ectopic pregnancy, with clear localization and vascular characteristics supporting the diagnosis.

At the initial presentation, the patient was hemodynamically stable, with a confirmed early ectopic pregnancy localized to the vaginal cuff. Management options discussed included the following:

• Immediate surgical intervention (exploratory laparotomy)
• Close inpatient observation with readiness for urgent surgery
• Counseling regarding the high risk of rupture due to the vascular nature of cuff implantation

The patient initially declined surgical intervention, necessitating a safety‐net approach. She was counseled regarding the following:

• The risk of rapid clinical deterioration, including hemorrhage and shock
• Warning signs requiring immediate return (abdominal pain, dizziness, and syncope)
• The need for urgent reassessment within a short interval

Despite this, the patient re‐presented 48 h later with hemodynamic instability and hemoperitoneum, illustrating the unpredictable and rapidly progressive nature of such ectopic implantations.

This aspect of the case highlights an important clinical lesson:

even in stable patients, vaginal cuff ectopic pregnancy carries a high risk of sudden rupture, and delayed intervention may result in life‐threatening hemorrhage.

## 4. Diagnostic Assessment

A systematic evaluation of alternative ectopic implantation sites was undertaken based on detailed transvaginal sonographic findings and intraoperative correlation.

Ovarian ectopic pregnancy was considered unlikely, as both adnexal regions were clearly visualized and showed no evidence of a gestational sac within or attached to the ovarian parenchyma. The left ovary appeared morphologically normal, with no surrounding echogenic ring or intra‐ovarian gestational structure.

Tubal ectopic pregnancy, including tubal stump implantation, was excluded due to the absence of any adnexal mass or tubal ring sign, and the clear spatial separation between the gestational sac and the left fallopian tube. Additionally, the right adnexa had been surgically removed at the time of prior hysterectomy, further reducing the likelihood of tubal origin.

Broad ligament ectopic pregnancy was ruled out as the gestational sac was not located within the lateral pelvic compartments, nor was it seen between the leaves of the broad ligament. Instead, it was confined to the midline at the vaginal vault.

Primary peritoneal implantation was considered less likely given the absence of a free‐floating gestational sac or implantation on peritoneal surfaces. The gestational sac demonstrated a fixed position with a well‐defined interface at the vaginal cuff, rather than attachment to peritoneal structures such as the bowel, omentum, or pelvic sidewall.

Importantly, the direct contiguity between the gestational sac and the vaginal cuff scar, combined with its midline location and absence of adnexal or peritoneal involvement, strongly supported the diagnosis of a vaginal cuff ectopic pregnancy.

This diagnosis was ultimately confirmed intraoperatively, where the gestational tissue was found localized to the vaginal cuff region, with no evidence of adnexal or peritoneal implantation.

### 4.1. Intraoperative Confirmation

Emergency exploratory laparotomy confirmed the diagnosis. Approximately 2.5 L of hemoperitoneum was evacuated.

A gestational sac containing fetal and placental tissue was identified in the midline pelvis, firmly attached to the superior aspect of the vaginal cuff scar, corresponding to the prior hysterectomy closure site. The sac was in direct continuity with the fibrous cuff tissue, without interposed peritoneum.

The implantation site was focal (approximately 2–3 cm), with superficial to moderate trophoblastic invasion into the cuff scar, associated with localized neovascularization. There was no evidence of deep invasion into adjacent organs, including the bladder or rectum.

Careful inspection of the cuff demonstrated intact closure without macroscopic dehiscence or identifiable fistulous tract.

Both the left ovary and fallopian tube stump were intact and clearly separate from the implantation site, with no evidence of adnexal involvement. No gestational tissue was identified within the adnexa, broad ligament, or peritoneal surfaces.

These findings confirm a primary vaginal cuff ectopic pregnancy, with direct implantation on the cuff scar and no alternative site of origin.

### 4.2. Histopathological Examination

**Figure 2:**
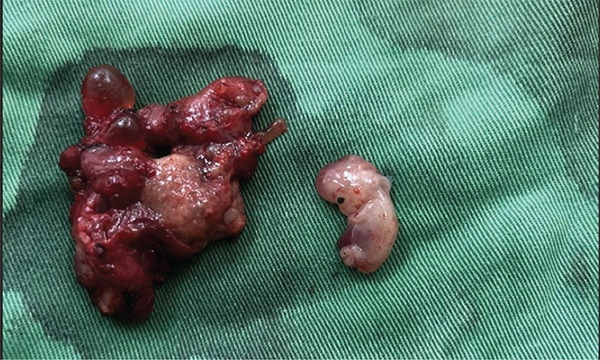
Gestational sac, placenta, and fetus following dissection.

The excised specimen (Figure 2), including the gestational sac, placental tissue, and fetal components, was submitted for detailed histopathological evaluation.

Microscopic examination demonstrated the following:

• Well‐formed chorionic villi with trophoblastic proliferation, confirming products of conception
• Presence of both cytotrophoblast and syncytiotrophoblast layers, consistent with viable early gestation
• Areas of hemorrhage and fibrin deposition, compatible with ectopic implantation
• No evidence of molar changes or malignant trophoblastic disease

These findings confirmed the diagnosis of ectopic pregnancy implanted outside the uterine cavity, correlating with intraoperative localization at the vaginal cuff.

### 4.3. Therapeutic Intervention

Due to worsening abdominal pain, hypotension, and ultrasonographic evidence of intraperitoneal bleeding, the patient underwent emergency exploratory laparotomy.

Upon entry into the abdominal cavity, approximately 2.5 L of hemoperitoneum was evacuated.

Careful exploration revealed a gestational sac attached to the vaginal cuff scar, located between the left ovary and the left infundibulopelvic ligament. The implantation site showed vascular trophoblastic tissue infiltrating the cuff scar, consistent with ectopic implantation.

No implantation was identified within the ovary, fallopian tube stump, or broad ligament.

The gestational sac and placental tissue were carefully dissected from the cuff, and the implantation base was ligated to achieve hemostasis.

‐Hemostasis secured.

‐Blood product resuscitation included the following:

Four units packed red blood cells.

Four units platelets.

Four units fresh frozen plasma.

### 4.4. Follow‐Up and Outcomes

The patient demonstrated progressive postoperative recovery following surgical intervention. Immediate postoperative care included close hemodynamic monitoring and transfusion support. Her vital signs stabilized within the first 24 h, with no further evidence of intra‐abdominal bleeding.

During hospitalization, the patient remained clinically stable, with no fever or signs of postoperative infection, normal wound healing, and gradual return to oral intake and mobility.

She was discharged in stable condition after appropriate recovery.

At subsequent follow‐up (2–4 weeks postoperatively), the patient remained asymptomatic, with no abdominal pain or vaginal discharge; physical examination was unremarkable; and no delayed complications (e.g., infection, bleeding, or pelvic collection) were identified.

She received counseling regarding the following:

‐The rarity of posthysterectomy pregnancy.

‐The potential mechanisms of occurrence.

‐The importance of early medical consultation in case of future symptoms.

## 5. Discussion

### 5.1. Summary of Key Findings

This case describes a rare posthysterectomy pregnancy implanted at the vaginal cuff, occurring 10 months after a total abdominal hysterectomy with right oophorectomy. Despite the absence of a uterus, the patient conceived, likely through ovulation from the preserved left ovary, followed by sperm passage via a microscopic or occult fistulous tract. Early TVUS confirmed a viable 8‐week embryo, but the patient later developed hemoperitoneum, requiring emergency laparotomy. Surgical excision of the gestational sac and massive transfusion led to a favorable outcome.

This case underscores the continued possibility of conception in reproductive‐aged women with retained ovarian function even after hysterectomy and highlights the importance of early recognition, prompt imaging, and timely surgical intervention.

### 5.2. Comparison With Existing Literature

Pregnancy after hysterectomy is exceptionally rare, with fewer than 100 cases reported worldwide, and only a subset involving implantation at the vaginal cuff. Published reports consistently identify two primary mechanisms:

1. Unrecognized early intrauterine pregnancy at the time of hysterectomy, or
2. Fistulous communication between the vaginal cuff and peritoneal cavity, permitting sperm to reach the ovary [7].

Similar to previous reports, this patient presented with pelvic pain that progressed to hypovolemic shock, an established hallmark of ruptured cuff implantation. The literature also supports that TVUS is superior to transabdominal imaging in detecting these highly atypical gestations, particularly in patients with altered pelvic anatomy [2].

Management strategies described globally align with this case: urgent surgical exploration, careful dissection to protect ureteral and ovarian structures, removal of gestational tissue, and aggressive resuscitation due to the highly vascular implantation site. Outcomes tend to be favorable when diagnosis and intervention occur promptly.

This case therefore reinforces established themes in the literature while adding additional evidence from a resource‐limited, high‐volume tertiary center in East Africa, an underrepresented setting in published reports.

### 5.3. Mechanisms of Posthysterectomy Pregnancy

Pregnancy following hysterectomy is an exceedingly rare but well‐documented phenomenon, with fewer than 100 cases reported in the literature. Several mechanisms have been proposed to explain this occurrence, including unrecognized early pregnancy at the time of hysterectomy, formation of a vaginal cuff peritoneal fistula, and transmigration of sperm or fertilized ovum through a residual fallopian tube or tubal stump [5].

Unrecognized early gestation at the time of hysterectomy is the most frequently reported mechanism in cases presenting shortly after surgery. In such situations, a fertilized ovum is already present prior to uterine removal and subsequently implants ectopically [5].

However, this mechanism is unlikely in our case, as the patient presented approximately 10 months after hysterectomy, making a pre‐existing pregnancy at the time of surgery implausible .

The second proposed mechanism involves the formation of a vaginal cuff–peritoneal fistula, which may allow spermatozoa to ascend from the vaginal vault into the peritoneal cavity, resulting in fertilization and subsequent ectopic implantation [6]. Such fistulas are often associated with postoperative complications, including infection, hematoma, or poor wound healing. In the present case, no clinical history of postoperative infection or cuff complications was documented, and importantly, no macroscopic fistulous tract was identified intraoperatively. Nevertheless, the absence of a visible tract does not definitively exclude this mechanism, as microscopic fistulous communications have been described and may not be detectable on imaging or surgical exploration [5].

The third mechanism, transmigration of sperm through a patent fallopian tube or tubal stump, is considered the most plausible explanation in delayed presentations such as this case. Following hysterectomy, if one or both fallopian tubes remain intact, sperm may ascend through the vaginal cuff, enter the peritoneal cavity, and reach the ovulated oocyte. In our patient, the left ovary was preserved and demonstrated evidence of recent ovulation, while the left fallopian tube stump was intact and uninvolved in implantation. This anatomical continuity supports the possibility of transperitoneal fertilization, followed by secondary implantation at a susceptible site.

The localization of implantation at the vaginal cuff scar further suggests that local tissue factors may play a contributory role. Scar tissue at the cuff may provide a site of reduced resistance with altered vascularity, facilitating trophoblastic invasion. In our case, both sonographic and intraoperative findings demonstrated direct attachment of the gestational sac to the cuff scar with surrounding trophoblastic vascularization, supporting this hypothesis.

Importantly, the absence of implantation within the ovary, fallopian tube stump, broad ligament, or peritoneal surfaces confirmed both on imaging and during laparotomy further strengthens the diagnosis of primary vaginal cuff ectopic pregnancy rather than secondary implantation from another site.

In summary, while multiple mechanisms have been described in the literature, the most plausible explanation in this case is transperitoneal migration of sperm through the preserved left adnexa, followed by implantation at the vaginal cuff, potentially facilitated by microscopic fistulous communication or scar‐related susceptibility. This case highlights the importance of considering delayed ectopic pregnancy even long after hysterectomy, particularly in patients with retained adnexal structures.

While medical management with methotrexate is well established for selected cases of tubal ectopic pregnancy, recent efforts have focused on improving patient selection through predictive models.

Yeniocak et al. developed a scoring system incorporating *β*‐hCG levels, ultrasonographic findings, and clinical parameters to predict treatment success with single‐dose methotrexate. Such approaches underscore the increasing emphasis on standardized and individualized care pathways in ectopic pregnancy [8]. However, in rare and anatomically complex presentations such as vaginal cuff pregnancy following hysterectomy, these models have limited applicability, and prompt surgical intervention remains the definitive treatment. Nevertheless, the principle of structured clinical evaluation remains highly relevant, particularly in guiding timely decision‐making and avoiding delays in high‐risk cases.

### 5.4. Strengths

Rarity and educational value: This case contributes to the scarce global data on vaginal cuff pregnancies posthysterectomy, especially from sub‐Saharan Africa, where such documentation is extremely limited.

Clear diagnostic imaging: Use of TVUS allowed precise localization and early confirmation of a viable ectopic gestation.

Detailed intraoperative findings: Careful description of surgical anatomy including the position between the infundibulopelvic ligament and ovary provides clinically relevant information for future surgeons managing similar cases.

Successful management in an emergency context: The case demonstrates that even with advanced hemoperitoneum, timely laparotomy and adequate transfusion support can result in excellent maternal outcomes.

### 5.5. Limitations

• Absence of prehysterectomy imaging or early postoperative evaluation, making it impossible to determine whether conception began before or after the hysterectomy.
• Lack of definitive identification of the fistulous tract, which is a common challenge reported in the literature.
• The patient initially declined surgical intervention, delaying definitive management and limiting early pathological evaluation.
• Single‐case limitation: As with all case reports, findings cannot be generalized and represent one patient′s unique clinical course.

### 5.6. Clinical Implications

This case reinforces several important clinical considerations:

‐Ectopic pregnancy must remain part of the differential diagnosis in reproductive‐aged women—with preserved ovarian tissue—even after total hysterectomy.

‐TVUS should be promptly performed in posthysterectomy patients with pelvic pain or a positive pregnancy test, as transabdominal scans may miss cuff implantations.

‐Early identification of hemodynamic compromise is critical, as these pregnancies carry a high risk of catastrophic intraperitoneal bleeding.

‐Surgical exploration must be undertaken without delay once instability or intraperitoneal fluid is detected, given the potential for rapid deterioration.

Clinicians should maintain a high index of suspicion for fistulous communication in patients presenting with unexpected reproductive symptoms after hysterectomy.

### 5.7. What This Case Adds to the Current Knowledge

‐Provides one of the very few documented cases of vaginal cuff ectopic pregnancy in a posthysterectomy patient from East Africa, contributing regional representation to an extremely scarce global literature.

‐Demonstrates that ovulation and fertilization remain possible even long after hysterectomy, emphasizing the need for patient counseling regarding residual pregnancy risk.

‐Highlights that TVUS remains the most reliable diagnostic tool in detecting these rare implantations.

‐Offers detailed intraoperative findings and management principles that can guide clinicians encountering similar emergencies, especially in low‐resource environments.

‐Reinforces the life‐saving role of rapid surgical intervention and massive transfusion protocols in managing hemorrhagic cuff pregnancies.

## 6. Patient Perspectives

The patient provided the following statement:

“I was shocked to learn that I was pregnant after having a hysterectomy. At first, I did not believe it was possible, and I felt confused and frightened when the doctors explained the risks. When my pain worsened, I was very scared, but the medical team acted quickly and saved my life. I am grateful for the care I received and for the way the doctors explained everything to me. This experience taught me the importance of seeking medical attention quickly whenever something does not feel right.”

## 7. Informed Consent and Ethical Consideration

Written informed consent was obtained from the patient for publication of this case report, including the accompanying clinical images. The patient was informed that the images would be used for scientific publication and educational purposes, and all identifying information has been removed to preserve confidentiality. A copy of the signed consent form is available for review by the journal upon request.

## Author Contributions

**Emmanuel Nshimiyumuremyi:** conceptualization, clinical management, data curation, methodology, writing – original draft, visualization, project administration, supervision. **Dawit Worku Kassa:** clinical management, investigation, methodology, writing – review and editing. **Bagambe Patrick:** surgical supervision, validation, resources; writing – review and editing. **Diomede Ntasumbumuyange:** surgical support, critical revision of the manuscript, supervision. **Fikremelekot Temesgen:** clinical assessment, data curation, writing – review and editing. **Thomas Ugiruwatuma:** patient follow‐up, data acquisition. **Viateur Mutabazi:** literature search, data verification. **Theogene Bazimaziki:** assistance with clinical data collection, visualization. **Emmanuel Itangishaka:** support during surgical intervention, resources, writing – review and editing. **Vedaste Musangamfura:** diagnostic imaging support, ultrasound interpretation, writing – review and editing. **Etienne Nsengimana:** pathology analysis, validation, writing – review and editing. **Oscar Umuhoza:** literature review, writing – review and editing; final manuscript preparation.

## Funding

No funding was received for this manuscript.

## Disclosure

All authors reviewed and approved the final version of the manuscript.

## Conflicts of Interest

The authors declare no conflicts of interest.

## Data Availability

All data supporting the findings of this case report are included within the article. Additional details are available from the corresponding author upon reasonable request, in compliance with patient confidentiality.
